# Effect of low TiO_2_ additions on the microstructure and mechanical properties of stir-cast Al–Zn composites

**DOI:** 10.1039/d6ra00784h

**Published:** 2026-03-25

**Authors:** Md. Sabbir Hossain Shawon, Aquib Rahman, Chowdhury Ahmed Shahed, Rezaul Karim Nayeem, Rafia Islam, Ashraf Ali Ratul, Md Zillur Rahman

**Affiliations:** a Department of Industrial and Production Engineering, Ahsanullah University of Science and Technology Dhaka 1208 Bangladesh; b Department of Mechanical Engineering, Ahsanullah University of Science and Technology Dhaka 1208 Bangladesh md.zillur.rahman.phd@gmail.com

## Abstract

Low-weight ceramic reinforcement offers an effective route to enhance aluminum-based metal matrix composites while preserving process scalability and cost efficiency. In this study, Al-2 wt% Zn composites reinforced with 0–0.5 wt% TiO_2_ particles were fabricated *via* conventional stir casting, and their microstructural evolution and mechanical performance were systematically investigated. Microstructural characterization (OM, SEM-EDS, and XRD) reveals that TiO_2_ additions promote significant grain refinement, improved phase homogeneity, and stable dispersion of TiO_2_/oxide-bearing regions within a continuous α-Al matrix, while Zn remains predominantly in solid solution. A progressive reduction in void content (from 0.98% to 0.62%) further indicates improved structural integrity with increasing TiO_2_ fraction. Mechanical testing demonstrates a substantial enhancement in performance with increasing reinforcement content. At 0.5 wt% TiO_2_, the composite exhibits improvements of approximately 52% in hardness, 32% in impact strength, and 23% in tensile strength compared to the unreinforced alloy due to Hall–Petch grain-boundary strengthening, Orowan-type particle strengthening, and effective load transfer between matrix and reinforcement. Fractographic analysis confirms a transition from predominantly ductile shear fracture in the matrix alloy to a mixed-mode fracture mechanism governed by particle-assisted void nucleation and crack deflection in the reinforced composites. Overall, the results demonstrate that very low TiO_2_ additions can deliver significant mechanical enhancement without the processing challenges associated with high reinforcement loadings, making Al–Zn–TiO_2_ composites potential candidates for lightweight structural applications in automotive and aerospace sectors.

## Abstract 2

0.5 wt% TiO_2_ refined grains reduce voids from 0.98% to 0.62% and increase hardness by 52%, impact strength by 32%, and tensile strength by 23% *versus* unreinforced Al–Zn.

## Graphical abstract

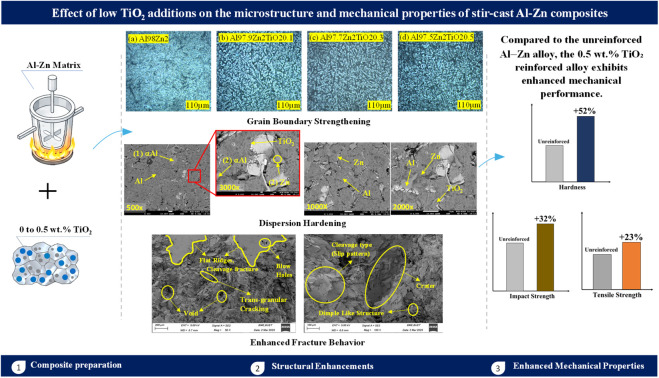

## 1 Introduction

Aluminum (Al) alloys are widely used in marine, automotive, and aerospace applications due to their low density, high strength-to-weight ratio, superior corrosion resistance, and recyclability.^1,2^ The global shift toward lightweight and energy-efficient structures has further accelerated research on Al alloys and their derivatives.^3,4^ Among these, Al–Mg–Si alloy systems are extensively utilized for lightweight structural components owing to their excellent formability and weldability.^5^ These alloys are particularly valued for their balanced strength, corrosion resistance, and thermal stability, largely attributed to the formation of a protective passive Al_2_O_3_ layer that resists oxidation in marine and humid environments.^6^

Owing to these favorable characteristics, pure Al and its alloys have become prominent matrix materials for the development of Al metal matrix composites (AMMCs). Incorporating reinforcements into Al matrices significantly improves mechanical strength, hardness, and wear resistance. Early studies primarily focused on single ceramic reinforcements, such as SiC and Al_2_O_3_, to enhance hardness and tribological performance.^7^ However, recent research has increasingly focused on hybrid reinforcement systems that synergistically improve multiple mechanical properties simultaneously.^8,9^ Such hybrid composites often exhibit superior performance while enabling near-net-shape fabrication, reduced machining requirements, and improved surface finish for wear-critical applications.^10^ As a result, hybrid Al-MMCs have emerged as promising candidates for next-generation lightweight structural materials. Given that Al alloys are already widely used in industry, incorporating suitable reinforcements provides an effective strategy to tailor their mechanical and tribological properties while maintaining manufacturing compatibility. Moreover, reinforced alloys can enhance manufacturing efficiency by enabling near-net-shape processing, thereby reducing machining time and minimizing secondary operations.^11^ In addition, the superior surface characteristics of MMCs can further decrease finishing requirements in wear-intensive applications.^12^

Ceramic and metallic reinforcements, particularly SiC and Al_2_O_3_, have been widely explored due to their hardness, thermal stability, and wear resistance.^13,14^ However, Al_2_O_3_ suffers from poor wettability and weak interfacial bonding with the Al matrix, necessitating surface modification or pretreatment.^15^ Boron carbide (B_4_C) exhibits high hardness and low density, making it suitable for armor applications, but it is costly and chemically reactive at elevated temperatures.^16^ Titanium dioxide (TiO_2_) has recently emerged as an attractive reinforcement because of its high hardness, good compatibility with Al, and cost-effectiveness.^17,18^ TiO_2_ additions have been shown to improve both hardness and corrosion resistance. Metallic reinforcements such as copper (Cu) and zinc (Zn) are also of interest. Cu can enhance strength and aging behavior,^19,20^ but often promotes galvanic corrosion.^21^ In contrast, Zn provides grain refinement and strength improvement with relatively uniform corrosion behavior due to its limited electrochemical potential difference with Al.^22^ These characteristics make Zn a promising alloying addition for Al-based composite systems.

Despite these benefits, single reinforcements can adversely affect other properties; for example, Cu reduces corrosion resistance. Lokesh *et al.*^23^ reported that integrating Cu and TiO_2_ enhanced both hardness and tensile strength in Al MMCs. Similarly, incorporating Si and Zn improved corrosion resistance.^24^ Hybrid systems such as Al + TiO_2_ + Cu demonstrated balanced mechanical and corrosion properties, where TiO_2_ mitigated the corrosion susceptibility induced by Cu.^25^ Building on these findings, TiO_2_–Zn hybrid reinforcement in an Al matrix is expected to provide an improved balance of strength, hardness, and corrosion resistance. Such a combination is particularly attractive for structural components used in marine and aerospace environments, where both durability and environmental stability are critical.

The weight fraction of reinforcements critically influences composite performance. The literature shows that small TiO_2_ additions (0.1–0.5 wt%) refine grain boundaries, thereby enhancing tensile strength, hardness, and corrosion resistance.^26^ However, higher TiO_2_ content may lead to clustering and reduce void volume, thereby degrading performance.^27^ Similarly, moderate Zn reinforcement (∼2 wt%) improves tensile strength, but excessive Zn can compromise corrosion resistance and distort the microstructure.^28^ Therefore, optimizing low-weight percentages of TiO_2_ and Zn can yield composites with balanced mechanical and electrochemical behavior. In a hybrid system, TiO_2_ can offset Zn's corrosion drawbacks, while Zn contributes to tensile strength, ductility, and structural stability.

Despite extensive research on Al-based MMCs, several important gaps remain. Many previous studies have focused on high reinforcement loadings (>5 wt%), expensive nanoparticles, or processing routes that are prone to agglomeration and limited in scalability.^29,30^ In contrast, the behavior of an Al-2 wt% Zn matrix reinforced with very low TiO_2_ additions (0.1–0.5 wt%) has received limited attention, particularly under conventional stir-casting conditions. The novelty of the present work lies in demonstrating that such low TiO_2_ additions can still produce measurable improvements in microstructural refinement, void reduction, hardness, impact strength, and tensile strength without the processing complexity associated with high reinforcement fractions. In this system, Zn acts as an alloying addition in the Al matrix, while TiO_2_ serves as the ceramic particulate reinforcement; therefore, the composite is more precisely described as a TiO_2_-reinforced Al–Zn composite rather than a broadly defined “hybrid reinforcement” system. Accordingly, the specific objective of this study is to determine how 0.1–0.5 wt% TiO_2_ additions influence the microstructure, density/void content, hardness, impact strength, tensile properties, and fracture behavior of stir-cast Al-2 wt% Zn composites.

## 2 Materials and methods

### 2.1 Materials

Commercially pure Al (≥99.5% purity) and Zn (≥99.9% purity) were procured from a certified industrial supplier in Dhaka, Bangladesh. TiO_2_ particles with an average particle size of approximately 1 µm and ≥99% purity were supplied by Millennium Dreams Co., Bangladesh. In the composite system, Al and Zn served as the matrix constituents, with Zn acting as a solid-solution strengthening alloying element. TiO_2_ particles were incorporated as ceramic reinforcements to promote grain refinement, dispersion strengthening, and microstructural stabilization. The TiO_2_ content was varied from 0.1 to 0.5 wt% to systematically evaluate its influence on the microstructural characteristics and mechanical properties of the composites. The composition details and nomenclature of the fabricated samples are summarized in Table 1.

**Table 1:** Composition and nomenclature of the fabricated composite samples

Sample code	Al (wt%)	Zn (wt%)	TiO_2_ (wt%)	Theoretical density (g cm^−3^)	Experimental density (g cm^−3^)	Void (%)
Al98Zn2	98%	2%	0%	2.734	2.707 ± 0.020	0.98
Al97.9Zn2TiO_2_0.1	97.9%	2%	0.1%	2.735	2.711 ± 0.009	0.87
Al97.7Zn2TiO_2_0.3	97.7%	2%	0.3%	2.737	2.717 ± 0.004	0.73
Al97.5Zn2TiO_2_0.5	97.5%	2%	0.5%	2.739	2.722 ± 0.001	0.62

### 2.2 Composite manufacturing

**Fig. 1:**
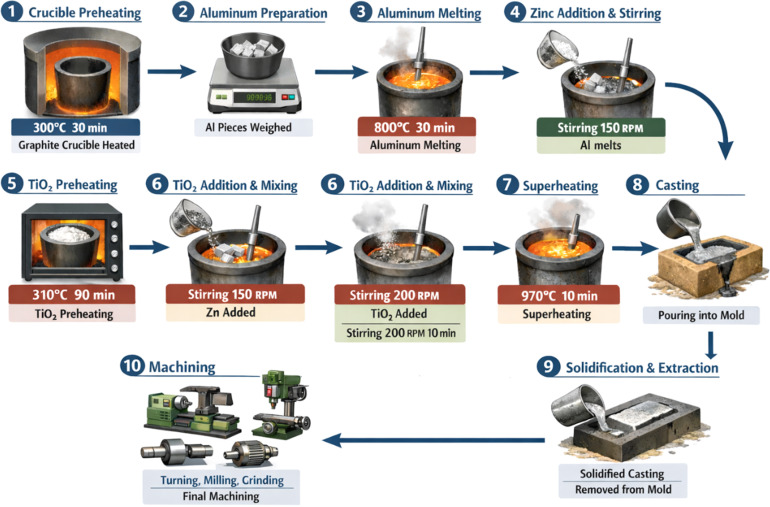
Schematic diagram of the manufacturing process of Al–Zn–TiO_2_ composite.

Fig. 1 shows the schematic diagram of the manufacturing process of the Al–Zn–TiO_2_ composites. The composites were fabricated using the stir-casting technique, which consisted of a graphite crucible, a gas-fired furnace, a mechanical steel stirrer, and a sand mold. Prior to melting, the graphite crucible was preheated in the furnace at 300 °C for 30 min to eliminate residual moisture. Al was cut into small pieces, accurately weighed using an electronic balance, and subsequently charged into the preheated crucible. The furnace temperature was gradually increased to 800 °C and maintained for 30 min to ensure complete melting of the Al. After the alloy reached a fully molten state, pure Zn was added to the melt, followed by mechanical stirring at 150 rpm for 5 min to promote uniform alloying. Stirring was carried out using a four-blade steel impeller positioned approximately two-thirds of the melt depth from the surface to ensure effective circulation of the molten metal. The impeller diameter was set to roughly one-third the crucible diameter, providing adequate shear flow while minimizing excessive turbulence. Separately, TiO_2_ reinforcement particles were preheated at 310 °C for 90 min to remove moisture and reduce the likelihood of oxidation. The preheated TiO_2_ particles were then gradually introduced into the molten Al–Zn alloy. To improve particle dispersion and compositional homogeneity, the stirring speed was increased to 200 rpm and maintained for 10 min, facilitating the formation of a uniform semi-solid slurry. During this process, a controlled vortex was generated to assist particle incorporation, while the particle addition rate was carefully regulated to minimize air entrapment. Subsequently, the melt temperature was raised to 970 °C and held for 10 min to improve wettability and ensure complete incorporation of the reinforcement particles. The molten composite was then poured into a preheated sand mold and allowed to solidify under ambient conditions. After complete solidification, the cast specimens were removed from the mold and subjected to machining operations, including turning, milling, and grinding, to eliminate surface irregularities and obtain specimens suitable for further characterization and testing. In total, three composite samples with varying TiO_2_ reinforcement contents and one unreinforced Al–Zn alloy sample were fabricated.

### 2.3 Characterization of composites

#### 2.3.1 Density measurement

The experimental densities of the Al98Zn2 matrix alloy and Al97.9Zn2TiO_2_*x* (*x* = wt%) composites were determined using Archimedes' principle in accordance with ASTM C373.^31^ A precision electronic balance (A&D ER-182A, A&D Company Ltd, Japan) was used to measure the specimen weight in air (*W*_a_) and while immersed in distilled water (*W*_w_). Distilled water with a density of 1.0 g cm^−3^ was used as the immersion medium. The experimental density (*ρ*_ce_) of each specimen was calculated using eqn (1):1
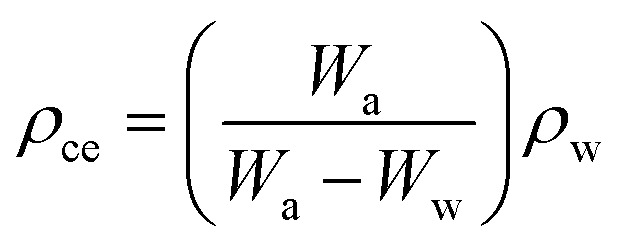
where *W*_a_ and *W*_w_ are the specimen weights in air and water, respectively, and *ρ*_w_is the density of the water. The theoretical densities (*ρ*_ct_) of Al98Zn2 and Al97.9Zn2TiO_2_*x* specimens were calculated using eqn (2):

2 *ρ*_ct_ = *v*_r_*ρ*_r_ + (1 − *v*_r_)*ρ*_m_

where *v*_r_ is the volume fraction of the reinforcement, *ρ*_r_ and *ρ*_m_ are the densities of the matrix (Al98Zn2) and reinforcement (TiO_2_). The void content (*V*_v_) of Al98Zn2 and Al97.9Zn2TiO_2_*x* composites can be estimated using eqn (3):3
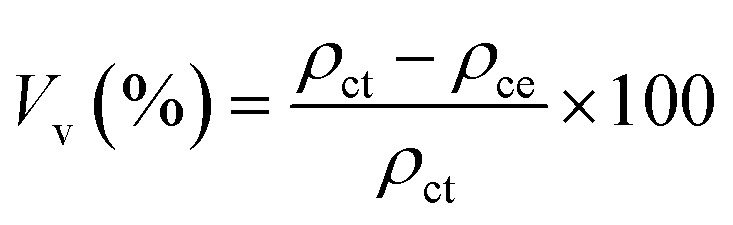


#### 2.3.2 Optical microscopy (OM) characterization

Optical microstructural analysis was performed using an optical microscope (OPTIKA-300, OPTIKA S.r.l., Italy) at 200× magnification with an eyepiece field number of 22 mm. Metallographic specimens with dimensions of 10 mm × 10 mm × 5 mm were prepared by sequential grinding using SiC emery papers of grit sizes P300, P600, P800, P1000, P1200, and P1500. Final polishing was performed using alumina slurry on a wet polishing cloth for 10 min to achieve a mirror-like surface. After polishing, specimens were cleaned and dried with acetone to remove residual contaminants and minimize surface oxidation prior to observation.

Grain size was quantified by the ASTM E112 line-intercept method.^32^ Optical micrographs of the polished specimens were analyzed using ImageJ software (National Institutes of Health, USA).^33^ Before measurement, the images were calibrated against the microscope reference scale, in which one minor division corresponded to 10 µm at 1000× magnification; the equivalent calibration factor was then applied to the 200× images in ImageJ. For clearer delineation of grain boundaries, the images were converted to grayscale before analysis. For each specimen, seven test lines with random orientations were superimposed on the micrograph, and the number of grain-boundary intersections along each line was counted. The mean intercept length was calculated according to ASTM E112 using eqn (4):

4 *l̄* = *L*_T_/*N*_T_

where *l̄* is the mean intercept length, *L*_T_ is the total test line length, and *N*_T_ is the total number of grain boundary intersections.

#### 2.3.3 X-ray diffraction (XRD) characterization

Phase identification was performed using an X-ray diffractometer (PANalytical Empyrean, Malvern Panalytical, Netherlands) equipped with a Cu Kα radiation source (*λ* = 1.5406 Å) and a Ni Kβ filter. The diffractometer was operated in the *θ*–2*θ* Bragg–Brentano geometry at an accelerating voltage of 60 kV and an appropriate tube current (30–45 mA) to achieve high signal-to-noise ratios. Diffraction data were collected using a high-resolution hybrid pixel detector (PixCel or equivalent). The XRD patterns were recorded over a 2*θ* range of 10–80° with a step size of 0.02° and a scan speed of 2° min^−1^. This scanning range enabled the reliable identification of all major crystalline phases present in the matrix and reinforced composite specimens.

#### 2.3.4 Scanning electron microscopy (SEM) characterization

The surface morphology and microstructural features of the composites were examined using a field-emission scanning electron microscope (JEOL JSM-7600F, JEOL Ltd, Tokyo, Japan). Specimens were mounted on Al stubs without conductive coating, as the metallic matrix provided sufficient electrical conductivity to prevent surface charging. Micro-images were then captured to evaluate reinforcement dispersion, particle–matrix interfacial bonding, and fracture-related features.

#### 2.3.5 Energy-dispersive X-ray spectroscopy (EDS) characterization

Elemental composition and phase distribution were analyzed using energy-dispersive X-ray spectroscopy (EDS) attached to the JEOL JSM-7600F FESEM. The composite's inherent conductivity enabled EDS analysis without additional surface coating. Elemental spectra and mapping were acquired to confirm the presence and spatial distribution of Zn and TiO_2_ reinforcements and to assess possible interfacial reactions within the composite matrix.

#### 2.3.6 Tensile test

Tensile tests were conducted in accordance with ASTM E8M using a universal testing machine (M500-50CT, Testometric Co. Ltd, Rochdale, UK) equipped with a 50 kN load cell.^34^ Cylindrical specimens with a diameter of 10 mm and gauge length of 50 mm were tested at a constant crosshead speed of 1 mm min^−1^. All tensile tests were conducted on at least five specimens per composition, and the reported values represent the mean results with corresponding standard deviations.

#### 2.3.7 Impact test

Charpy impact testing was conducted in accordance with ASTM E23 using a Charpy impact tester (QPI-IC-21J, Qualitest FZE, UAE).^35^ Standard V-notched specimens with dimensions of 56 mm × 10 mm × 10 mm were machined from the fabricated composites. A pendulum with a maximum capacity of 21 J was released from the standard initial angle (approximately 150°), and the absorbed impact energy was recorded digitally. For each composition, at least five specimens were tested to ensure repeatability. The reported impact strength values represent the average absorbed energy per unit cross-sectional area (kJ m^−2^) and the corresponding standard deviation.

#### 2.3.8 Hardness test

Brinell hardness measurements were conducted in accordance with ASTM E10 using a Brinell hardness tester (FB-3000LC, Future-Tech Corp., Kawasaki, Japan).^36^ Rectangular specimens with dimensions of 25 mm × 25 mm × 6 mm were evaluated using a 5 mm diameter tungsten carbide ball indenter under an applied load of 250 kgf with a dwell time of 10 s. For each composition, five independent indentations were performed at different locations on the specimen surface to minimize the influence of local microstructural variations. The reported Brinell Hardness Number (BHN) values represent the average of five measurements along with the corresponding standard deviation. The Brinell Hardness Number (BHN) was calculated using eqn (5):5
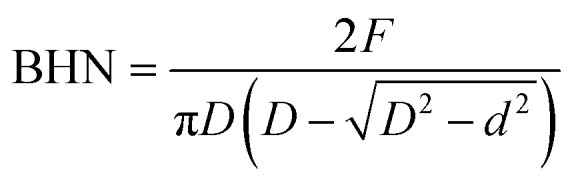
where *F* is the applied load (kgf), *D* is the diameter of the ball indenter (mm), and *d* is the average diameter of the indentation (mm).

## 3 Results and discussion

### 3.1 Microstructural analyses

#### 3.1.1 Optical microscopy analysis

**Fig. 2:**
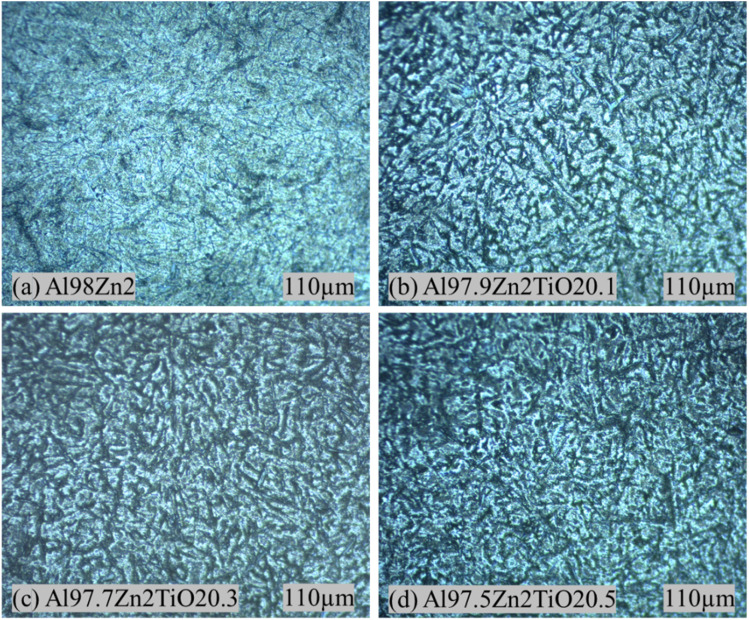
Optical micrographs of Al–Zn–TiO_2_ composites with varying TiO_2_ contents: (a) 0 wt%, (b) 0.1 wt%, (c) 0.3 wt%, and (d) 0.5 wt% TiO_2_.

The optical micrographs in Fig. 2 compare the Al-2 wt% Zn alloy (Fig. 2a) with Al-2 wt% Zn reinforced by increasing TiO_2_ contents from 0.1 to 0.5 wt% (Fig. 2b–d). The unreinforced Al-2 wt% Zn sample exhibits a relatively coarser cellular or dendritic morphology, whereas TiO_2_ addition produces a progressively finer and more uniformly distributed microstructural network across the field of view. This trend is consistent with the general behavior of ceramic-particle-reinforced Al composites processed by stir casting, where processing conditions and reinforcement incorporation strongly affect solidification morphology and defect population.^37^

The role of Zn in this system is primarily strengthening through a dilute substitutional solid solution rather than marked grain refinement. In binary Al–Zn alloys, Zn contributes to strengthening by interacting with dislocations through solute-dislocation interactions and associated local elastic and modulus mismatches, which increase resistance to plastic flow.^38,39^ The solute elements influence grain size mainly through growth-restriction effects and nucleation-related phenomena, and not all solutes provide strong refinement under typical casting conditions.^40^ This supports attributing the dominant refinement in Fig. 2b–d to the ceramic addition rather than to Zn. By contrast, the refinement observed with increasing TiO_2_ content is consistent with TiO_2_ acting as a heterogeneous nucleation aid and as a physical barrier to grain growth during solidification. The grain-refinement literature for cast Al highlights that the presence, potency, and number density of heterogeneous nucleation particles strongly govern their final grain size, particularly when particles have favorable nucleation characteristics and are sufficiently dispersed in the melt.^41^ Experimental studies on stir-cast TiO_2_-reinforced Al alloys similarly report a transition toward finer, more equiaxed grains with increasing TiO_2_ content, attributed to enhanced nucleation and constrained growth.^42^ The comparatively denser and more homogeneous morphology in Fig. 2c and d is therefore consistent with an increased population of nucleant particles and reduced effective grain–growth length scales.

Quantitative grain-size measurements obtained by the ASTM E112 line-intercept method are presented in Table 2. The base Al98Zn2 alloy exhibits a mean intercept length of 20.86 µm. With TiO_2_ addition, the mean intercept length decreases to 17.04 µm for Al97.9Zn2TiO_2_0.1, 16.38 µm for Al97.7Zn2TiO_2_0.3, and 14.85 µm for Al97.5Zn2TiO_2_0.5. This gradual decrease confirms progressive grain refinement with increasing TiO_2_ content and agrees well with the optical observations. At the highest TiO_2_ fraction (0.5 wt%), the microstructure remains fine but shows regions of locally intensified contrast, which can indicate incipient reinforcement clustering or local solidification heterogeneity. Such features are commonly reported in stir-cast MMCs when particle wetting and dispersion are imperfect, and they can coexist with overall grain refinement. This interpretation is aligned with the stir-casting process-structure sensitivity described in prior MMC studies.^37^ Overall, the OM results indicate that Zn mainly strengthens the matrix through solid-solution effects, whereas TiO_2_ is the principal factor governing grain refinement and microstructural homogenization. This refined microstructure is expected to contribute directly to the mechanical properties discussed in Section 3.2.

**Table 2:** Quantitative grain-size estimation of Al–Zn–TiO_2_ composites using the ASTM E112 line-intercept method

Al98Zn2	Line no.	1	2	3	4	5	6	7	Σ*L*/Σ*N*
	Line length (*L*_i_) (µm)	123.61	122.35	184.75	120.51	170.47	165.79	197.11	
	Number of intercepts (*N*_i_)	6	4	9	8	6	8	11	
	Intercept length (*L*_i_/*N*_i_) (µm)	20.60	30.58	20.52	15.06	28.41	20.72	17.92	20.86
Al97.9Zn2TiO_2_0.1	Line length (*L*_i_) (µm)	178.09	145.80	177.78	155.96	128.79	142.19	161.66	Σ*L*/Σ*N*
	Number of intercepts (*N*_i_)	10	7	13	8	8	8	10	
	Intercept length (*L*_i_/*N*_i_) (µm)	17.81	20.83	13.68	19.50	16.10	17.77	16.17	17.04
Al97.7Zn2TiO_2_0.3	Line length (*L*_i_) (µm)	179.09	233.85	242.30	204.79	171.33	178.66	263.77	Σ*L*/Σ*N*
	Number of intercepts (*N*_i_)	10	15	17	9	11	13	15	
	Intercept length (*L*_i_/*N*_i_) (µm)	19.90	15.59	14.25	22.75	17.13	13.74	17.58	16.38
Al97.5Zn2TiO_2_0.5	Line length (*L*_i_) (µm)	219.83	243.82	216.71	207.40	213.52	197.40	156.86	Σ*L*/Σ*N*
	Number of intercepts (*N*_i_)	15	18	15	14	14	12	10	
	Intercept length (*L*_i_/*N*_i_) (µm)	14.66	13.55	14.45	14.81	15.25	16.45	15.69	14.85

#### 3.1.2 XRD analysis

**Fig. 3:**
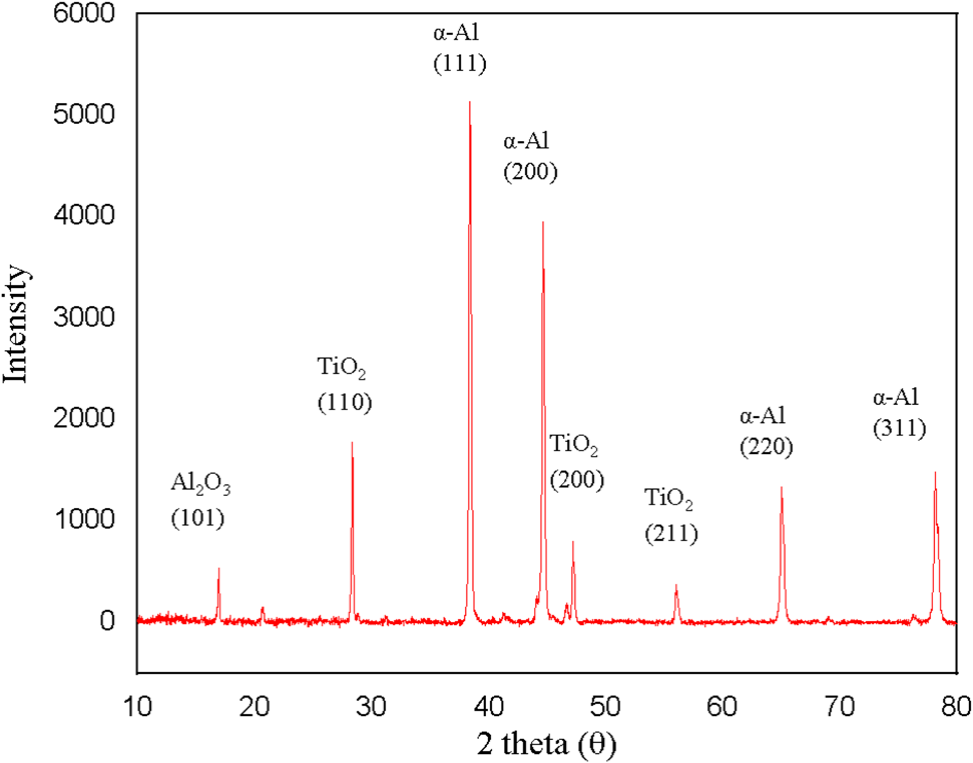
XRD pattern of the Al–Zn–TiO_2_ composite (10–80° 2*θ*). Peaks correspond to α-Al (FCC) and weak reflections from TiO_2_, with a trace Al–O peak (indexed as Al_2_O_3_).

Fig. 3 shows the XRD pattern of the Al–Zn–TiO_2_ composite. The diffractogram is dominated by the FCC (face-centered cubic) α-Al matrix, exhibiting intense reflections at 2*θ* ≈ 38.4°, 44.7°, 65.1°, and 78.2°, indexed to the (111), (200), (220), and (311) planes, respectively, consistent with standard α-Al diffraction positions.^43,44^ In addition, weak reflections attributable to TiO_2_ appear at ≈28°, ≈48°, and ≈55–56°, confirming retention of the ceramic reinforcement. Because TiO_2_ peaks are intrinsically weak at low reinforcement fractions and may partially overlap with intense α-Al reflections, polymorph identification should be treated cautiously; rutile is commonly reported near 27.4°, 36.1°, and 54.3°, whereas anatase typically exhibits peaks near 25.3°, 37.7–37.8°, and 48.0°.^45^ A trace Al–O-related reflection (labeled Al_2_O_3_) is also observed, which may arise from surface oxidation and/or limited interfacial reaction in Al/TiO_2_ systems where Al can reduce TiO_2_ under suitable thermal conditions, producing Al_2_O_3_-containing products.^46^ No distinct Zn-rich or Al–Zn intermetallic peaks are detected within the measurement sensitivity. This is consistent with Zn being largely accommodated in α-Al solid solution; notably, 2 wt% Zn corresponds to ∼0.83 at% Zn, close to the reported room-temperature solubility of Zn in α-Al (∼0.85 at% at 298 K), and any remaining Zn-containing precipitates would likely be below the XRD detection limit.^47^

#### 3.1.3 SEM analysis

**Fig. 4:**
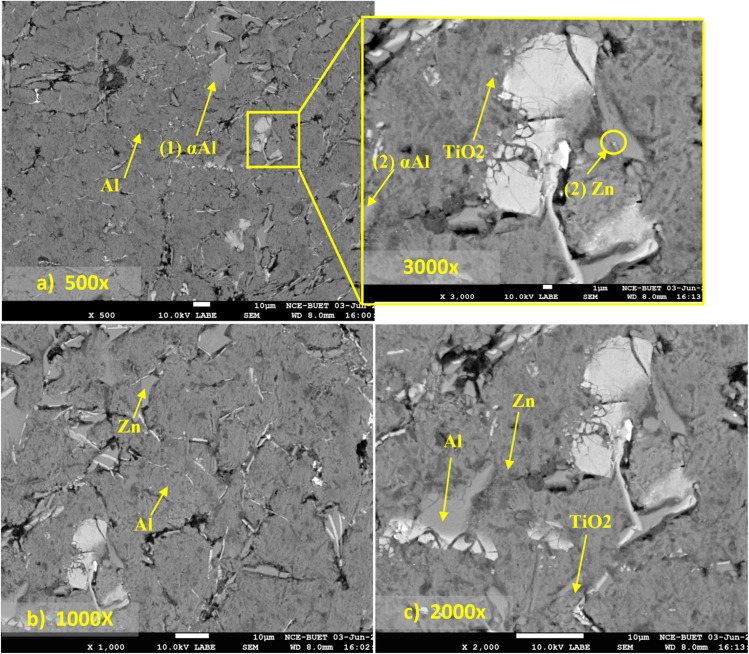
SEM micrographs of the Al-2 wt% Zn-0.5 wt% TiO_2_ composite at different magnifications: (a) 500× (3000× image placed at the inset), (b) 1000×, and (c) 2000× showing the α-Al matrix, TiO_2_/oxide-rich bright features, and interfacial voids/gaps.

Fig. 4 shows SEM micrographs of the Al-2 wt% Zn-0.5 wt% TiO_2_ composite at increasing magnification. This composition contains the highest TiO_2_ addition among the fabricated materials and therefore provides the most stringent assessment of reinforcement-related heterogeneity. The microstructure is dominated by a continuous α-Al matrix (dark-to-mid grey), within which brighter, angular to flake-like features are dispersed (Fig. 4a–c). In SEM imaging, grayscale intensity increases with the backscattered-electron coefficient and therefore correlates strongly with the mean atomic number and, to a lesser extent, with crystallographic and topographic contributions; consequently, Zn-enriched regions (with an atomic number higher than Al) and Ti-containing oxides/ceramics appear brighter than the α-Al matrix.^48^ This interpretation is also consistent with reported contrast and constituent morphologies in Al–Zn systems.^49,50^

At low magnification (Fig. 4a), the bright TiO_2_/oxide-rich features appear broadly distributed but with localized clustering, indicating incomplete deagglomeration during melt processing. Such clustering is commonly attributed to the poor intrinsic wettability of ceramic particulates by molten Al, particle–particle attraction, and the hydrodynamic limitations of vortex-based stirring, which collectively promote non-uniform dispersion, particularly as reinforcement content increases.^51^ At higher magnification (Fig. 4c), interfacial voids/gaps are evident adjacent to some bright particles and along particle-matrix boundaries. These discontinuities are most plausibly linked to incomplete wetting and interfacial gas entrapment during stirring, compounded by solidification shrinkage and thermal-expansion mismatch between the Al and ceramic phases, which can promote local debonding or microcrack initiation upon cooling.^52^ Overall, the images indicate a largely continuous α-Al matrix containing Zn-enriched and TiO_2_/oxide-rich constituents, while also highlighting reinforcement-induced heterogeneities (clusters and interfacial voids).

#### 3.1.4 EDS and elemental mapping analysis

**Fig. 5:**
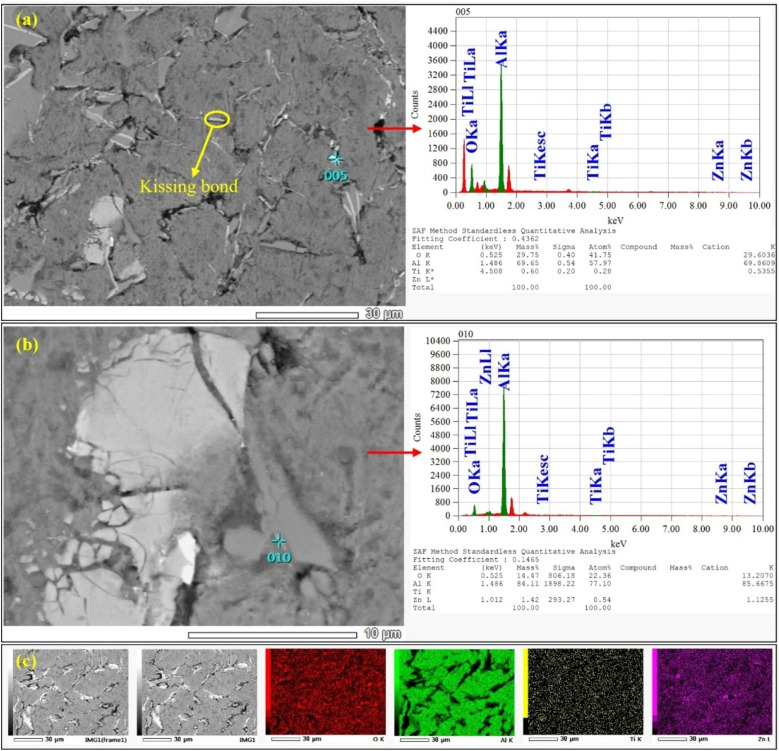
(a) SEM micrograph of the Al-2 wt% Zn-0.5 wt% TiO_2_ composite showing a kissing-bond-like interfacial discontinuity; corresponding point EDS spectrum (spot 005). (b) Higher-magnification SEM image near a bright oxide/ceramic-rich region with point EDS spectrum (spot 010). (c) Representative EDS elemental maps (O, Al, Ti, Zn) showing diffuse Zn distribution within the α-Al matrix and localized Ti–O enrichment associated with bright features.

Fig. 5 combines SEM imaging with point EDS and elemental mapping for the Al-2 wt% Zn-0.5 wt% TiO_2_ composite. The Zn map shows a broadly diffuse signal across the α-Al matrix (Fig. 5c), and the point spectra contain Zn peaks without indicating a dominant, continuous Zn-rich constituent at the micron scale (Fig. 5a and b). This distribution is consistent with the Al–Zn binary system, where Zn exhibits substantial solubility in α-Al at elevated temperatures and a reported room-temperature solubility of ∼0.85 at% (approximately 2 wt%).^53^ Because conventional SEM-EDS integrates X-rays over a micrometer-scale interaction volume in bulk specimens, chemically sharp segregation at submicron length scales (*e.g.*, nanoscale clustering or fine inter-dendritic enrichment) can remain undetected even when present.^54^

The Ti and O maps (Fig. 5c) exhibit weaker but distinct Ti–O enrichment associated with the brighter, angular, or flake-like regions in the SEM images (Fig. 5a and b), indicating TiO_2_-containing clusters and/or Ti-bearing oxide products. The comparatively low Ti intensity is expected at this reinforcement level because (i) TiO_2_ occupies a small local volume fraction, (ii) the EDS interaction volume dilutes the signal when particles are smaller than, or comparable to, the excitation volume, and (iii) any particle agglomerates can be partially masked by surrounding α-Al.^54^ The oxygen signal is spatially widespread, which is consistent with the inevitability of native alumina formation on molten Al and the presence of oxide-bearing features; however, its localization at bright features supports a microstructural origin rather than a purely superficial artifact (Fig. 5c).

Higher-magnification imaging reveals interfacial discontinuities adjacent to some bright features, including planar, intimate-contact gaps labeled as a “kissing bond” (Fig. 5a). Such kissing-bond-like interfaces are characteristic of incomplete metallurgical bonding caused by oxide films that remain trapped at an interface and prevent true wetting and coalescence.^55^ In Al processing, surface oxide films can be folded and entrained during turbulent filling or vigorous stirring, forming bi-films that behave as pre-existing cracks and readily open during solidification and straining.^56^ This mechanism provides a physically consistent explanation for the observed unbonded interfaces and associated voids near reinforcement-rich regions. Poor wettability of ceramic surfaces by molten Al, density mismatch, and melt-handling conditions promote particle clustering and localized voids, particularly as reinforcement content increases.^57^ These processing-driven heterogeneities can concentrate oxide films and trapped gas within reinforcement-rich zones, increasing the likelihood of interfacial gaps and void formation.^58^

The Al–O response in EDS, together with minor oxide indications from diffraction, can therefore be rationalized by two concurrent contributions: (i) native oxidation and bi-film-related oxide-film entrainment during melt processing, and (ii) partial chemical interaction between Al and TiO_2_. Reaction routes in Al–TiO_2_ systems are widely reported to produce Al_2_O_3_ along with Ti-containing reaction products, and *in situ* Al_3_Ti–Al_2_O_3_ formation from TiO_2_ and Al has been demonstrated under reactive processing conditions.^59^ Independent molten-Al studies also report Al_2_O_3_ generation *via* reaction with TiO_2_ in the 700–800 °C range under controlled conditions, although the extent of reaction is highly sensitive to time, atmosphere, particle size, and dispersion state.^60^ Accordingly, in the present composite, the most plausible interpretation is residual TiO_2_ together with a limited fraction of Ti-bearing oxides and reaction products rather than complete conversion to Al_2_O_3_.

Finally, the microstructure provides a mechanistic basis for the mechanical trends. Zn in solid solution and hard TiO_2_/oxide-bearing regions are expected to increase resistance to local plastic deformation and indentation, while pores and kissing-bond-like interfaces should reduce ductility and tensile reliability by serving as preferential crack-initiation sites.^61^ This is consistent with broader evidence that increasing the defect area fraction elevates local triaxial stress and accelerates ductile fracture in cast Al alloys.^62^ Reports on Zn-modified Al matrix composites similarly attribute improvements in strength and hardness to solid-solution and microstructural effects, provided defect control is maintained.^63^

### 3.2 Mechanical properties and failure analysis

#### 3.2.1 Tensile properties

**Fig. 6:**
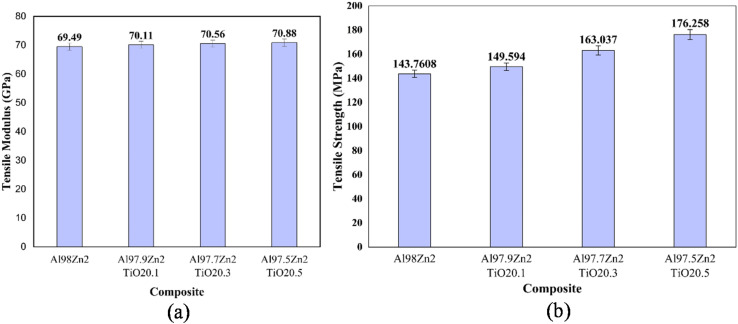
(a) Tensile modulus and (b) tensile strength of different Al–Zn–TiO_2_ composites.

The tensile modulus and tensile strength of the Al–Zn–TiO_2_ composites with increasing TiO_2_ contents are illustrated in Fig. 6. The unreinforced Al98Zn2 alloy shows the lowest tensile modulus of 69.49 GPa, which increases progressively to 70.11 GPa (Al97.9Zn2TiO_2_0.1), 70.56 GPa (Al97.7Zn2TiO_2_0.3), and 70.88 GPa (Al97.5Zn2TiO_2_0.5) (Fig. 6a). Overall, the modulus increases by approximately 2% at 0.5 wt% TiO_2_. This increase is consistent with TiO_2_'s higher intrinsic stiffness relative to Al, indicating that microstructural constraints and load-transfer mechanisms influence the composite's effective elastic response. In particulate metal matrix composites, the incorporation of stiff ceramic particles restricts matrix deformation and enhances the material's overall elastic stiffness. Additionally, the presence of finely dispersed TiO_2_ particles can enhance load transfer at the matrix–particle interface, thereby improving the composite's resistance to elastic deformation. These observations align with established micromechanical strengthening behavior in particle-reinforced Al matrix composites.^64^

In contrast to the modulus trend, the tensile strength increases with TiO_2_ content (Fig. 6b), from 143.76 MPa (Al98Zn2) to 149.59 MPa (0.1 wt% TiO_2_), 163.04 MPa (0.3 wt% TiO_2_), and 176.26 MPa (0.5 wt% TiO_2_), yielding a maximum improvement of 22.6%. This strengthening is consistent with (i) grain refinement, where TiO_2_ can promote finer grains by acting as nucleation sites and by limiting grain growth through particle pinning, and (ii) dislocation-based strengthening from dispersed particles. Hall–Petch boundary strengthening is widely validated for Al across relevant grain-size regimes, so any refinement directly increases yield and tensile strength by increasing boundary density that impedes dislocation motion.^65^ These mechanisms align with reports on TiO_2_-modified Al alloy that attribute strength gains to microstructural refinement and reinforcement-driven hardening.^66^

The dispersed TiO_2_ phase can further increase strength through Orowan bypassing and related particle–dislocation interactions, which raise the stress required for plastic flow when particle spacing is sufficiently small.^67^ Zn in solid solution also contributes to strengthening the Al matrix, complementing particle-related mechanisms. If, as indicated by density and void content in Table 1, TiO_2_ addition reduces casting defects, this will additionally improve tensile strength by reducing void-driven crack initiation, a well-known limitation in cast Al alloys.^68^ This combined evidence explains why strength can increase even when the apparent modulus decreases: stiffness is dominated by elastic load transfer and interfacial integrity, whereas tensile strength is governed by plasticity-controlled mechanisms such as grain refinement, particle–dislocation interactions, and defect suppression. Overall, Al97.5Zn2TiO_2_0.5 exhibits the highest tensile strength and improved structural integrity, although the increase in elastic stiffness is marginal. This composition is therefore promising for applications where strength and damage tolerance are prioritized over initial rigidity, whereas intermediate TiO_2_ contents may be preferable when stiffness is a primary design consideration.

##### 3.2.1.1 Tensile stress–strain curves

**Fig. 7:**
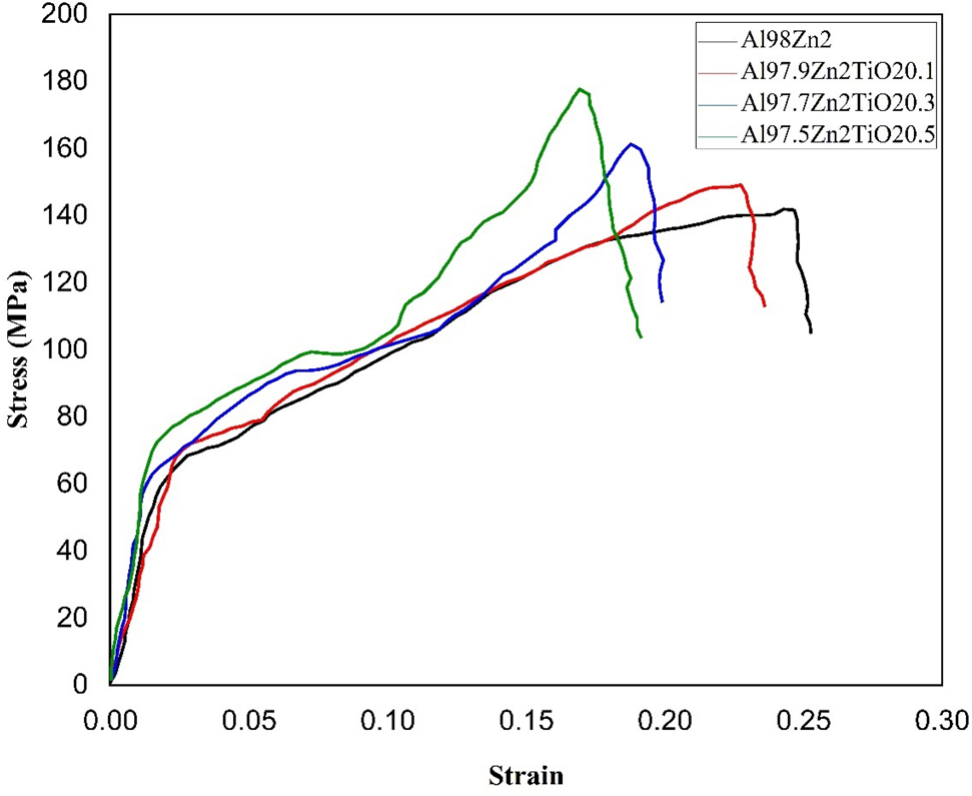
Tensile stress–strain curves of different Al–Zn–TiO_2_ composites.

Fig. 7 shows the tensile stress–strain curves of the Al-2 wt% Zn matrix and TiO_2_-reinforced composites. All compositions exhibit a typical ductile response consisting of (i) an initial quasi-linear elastic segment, followed by (ii) plastic flow with strain hardening up to the UTS (ultimate tensile strength), and then (iii) a post-UTS drop associated with necking and final fracture. The Al98Zn2 curve reaches a UTS of approximately 143 MPa and shows the highest elongation to fracture (about 0.25). The relatively extended plastic region indicates substantial uniform deformation prior to instability, consistent with the lower strength but higher ductility expected for the unreinforced matrix. With TiO_2_, the stress increases, and the UTS rises progressively to about 150 MPa (0.1 wt% TiO_2_), 163 MPa (0.3 wt% TiO_2_), and 175 MPa (0.5 wt% TiO_2_). This strengthening is due to load sharing, increased dislocation density arising from thermal-expansion mismatch during cooling, and Orowan-type dislocation bypassing when particle spacing is sufficiently small.^69^ The presence of these mechanisms typically increases strain hardening and elevates the stress required to maintain plastic flow, consistent with the higher peak stresses observed for the 0.3 and 0.5 wt% TiO_2_ curves. Similar strengthening with TiO_2_ additions in cast Al composites has been reported in the study.^70^

Despite higher UTS, the fracture strain decreases with increasing TiO_2_ content, falling from ∼0.25 (matrix) to ∼0.22 (0.1 wt% TiO_2_), ∼0.21 (0.3 wt% TiO_2_), and ∼0.19 (0.5 wt% TiO_2_). This strength-ductility trade-off is common in particle-reinforced Al systems because particles can introduce local strain incompatibility and stress concentrations that promote earlier damage initiation through particle cracking, interface decohesion, or void nucleation at particle clusters.^71^ Accordingly, the sharper terminal stress drop in the reinforced curves is owing to accelerated damage accumulation and final fracture after reduced post-uniform deformation, rather than as evidence of intrinsically brittle behavior. Overall, TiO_2_ additions substantially improve load-carrying capacity and peak tensile stress, while progressively reducing elongation to fracture.

##### 3.2.1.2 Fractography

**Fig. 8:**
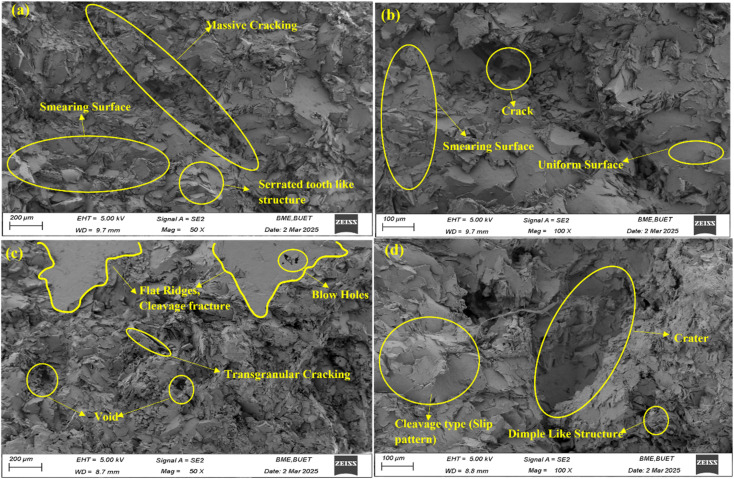
SEM fractography of tensile-fractured specimens showing morphological differences at two magnifications: (a and b) unreinforced Al98Zn2 alloy and (c and d) 0.5 wt% TiO_2_ reinforced Al97.5Zn2TiO_2_0.5 composite. Images (a and c) correspond to 50× magnification, highlighting overall fracture morphology, while (b and d) correspond to 100× magnification, revealing micro-scale fracture features such as shear flow, cleavage facets, voids, and dimples.

The SEM fracture surfaces provide insight into the failure mechanisms of the unreinforced alloy and the TiO_2_-reinforced composite at two different magnifications (Fig. 8). At low magnification (50×), the unreinforced Al98Zn2 alloy (Fig. 8a) shows extensive smearing and shear-flow features with long interconnected cracks, indicating dominant ductile shear deformation and crack coalescence after significant plastic flow. The stepped “serrated tooth-like” morphology suggests localized shear banding during the final stages of deformation, which is commonly associated with strain localization in Al alloys containing mobile solutes.^72,73^ These features are consistent with the high ductility and lower tensile strength observed for the matrix alloy. At higher magnification (100×), the same alloy surface (Fig. 8b) reveals relatively uniform deformation zones with localized cracks, confirming that failure occurred primarily through plastic shear and microvoid coalescence, characteristic of ductile fracture in cast Al alloys. In contrast, the 0.5 wt% TiO_2_ composite (Al97.5Zn2TiO_2_0.5) exhibits a distinctly different morphology. At 50× magnification (Fig. 8c), the fracture surface contains flat cleavage-like facets, ridges, and *trans*-granular cracks, together with voids and blow-hole defects. These features indicate the presence of localized brittle fracture regions superimposed on ductile deformation, suggesting that reinforcement particles act as stress concentrators and damage initiation sites.^74–76^ At 100× magnification (Fig. 8d), dimple-like depressions and crater-type features become visible, which are associated with particle pull-out, interfacial decohesion, and micro void nucleation around reinforcement particles.^77^ Such particle-controlled damage mechanisms promote void growth and crack propagation in particle-rich regions, leading to a mixed ductile–quasi-cleavage fracture mode. These morphological differences directly explain the mechanical behavior reported in Section 3.2.1. The ductile shear fracture of the unreinforced alloy enables larger plastic deformation, while the reinforced composite exhibits higher strength but reduced elongation due to particle-assisted void nucleation, interfacial debonding, and *trans*-granular crack propagation.^78–80^ Thus, the fractography confirms that TiO_2_ reinforcement enhances load-bearing capacity while simultaneously increasing damage sensitivity.

#### 3.2.2 Impact strength

**Fig. 9:**
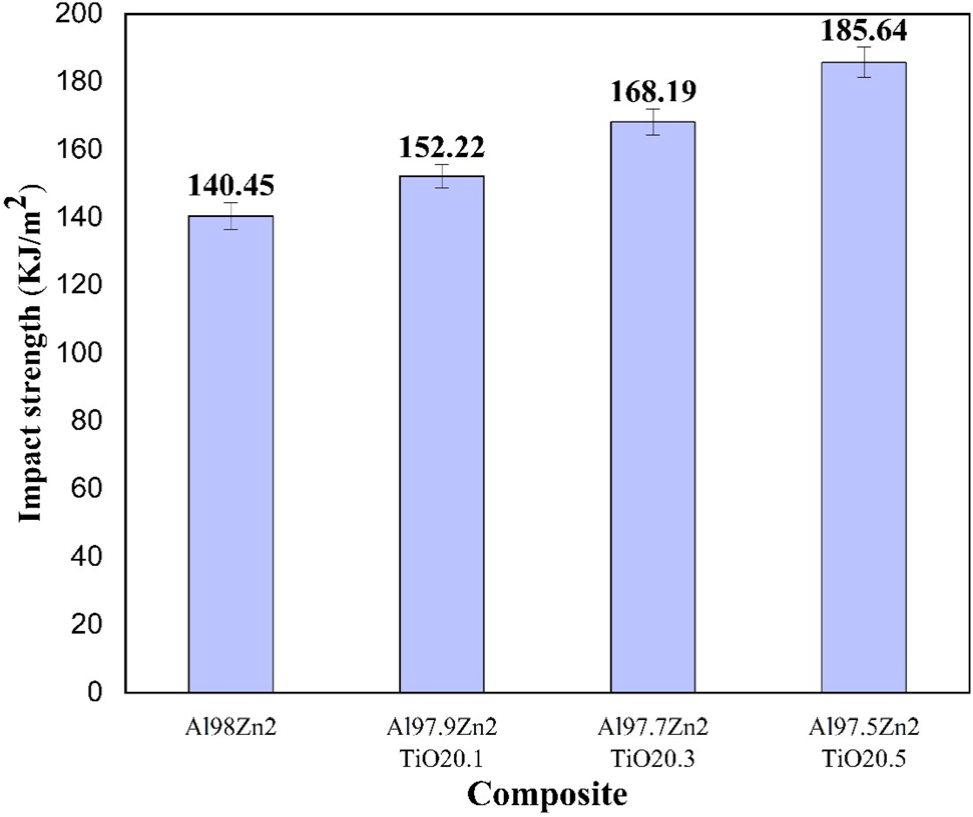
Impact strength of different Al–Zn–TiO_2_ composites.

Fig. 9 shows a progressive increase in impact strength with TiO_2_ addition to the Al-2 wt% Zn matrix. The unreinforced Al98Zn2 alloy exhibits the lowest impact strength (140.45 kJ m^−2^). The addition of 0.1 wt% TiO_2_ increases the value to 152.22 kJ m^−2^, while 0.3 wt% TiO_2_ produces a more significant improvement to 168.19 kJ m^−2^. The highest impact strength is achieved at 0.5 wt% TiO_2_ (Al97.5Zn2TiO_2_0.5), reaching 185.64 kJ m^−2^, corresponding to an increase of approximately 32% compared with the matrix alloy. The relatively small error bars indicate good repeatability and stable processing conditions across all compositions. The enhancement in impact performance is attributed to reinforcement-driven toughening and strengthening mechanisms that increase the energy required for crack initiation and propagation. In particulate-reinforced Al systems, well-dispersed ceramic particles promote dislocation accumulation and impede dislocation motion (including Orowan-type bypassing), thereby increasing strain hardening and plastic work during impact loading.^81^ TiO_2_ particles may also act as heterogeneous nucleation sites, refining the matrix microstructure and increasing barriers to slip and crack propagation. These effects align with previous studies reporting improved energy absorption in TiO_2_-modified Al alloys when particle dispersion and matrix–particle bonding are adequate. Additionally, Zn additions enhance matrix ductility and impact energy, complementing particle-induced strengthening and contributing to overall toughness.^82^ The significant increase in impact strength between 0.3 and 0.5 wt% TiO_2_ suggests that, within this range, the composite achieves a more favorable combination of particle dispersion, spacing, and interfacial bonding, leading to greater crack-path tortuosity and higher plastic energy dissipation before fracture. Similar improvements have been reported in stir-cast Al composites, where uniform reinforcement distribution and strong interfacial bonding inhibit crack growth and enhance impact resistance.^83^ Overall, the results demonstrate that TiO_2_ additions up to 0.5 wt% significantly improve the impact energy absorption of the Al–Zn–TiO_2_ system by promoting plastic deformation and suppressing rapid crack propagation.

#### 3.2.3 Hardness

**Fig. 10:**
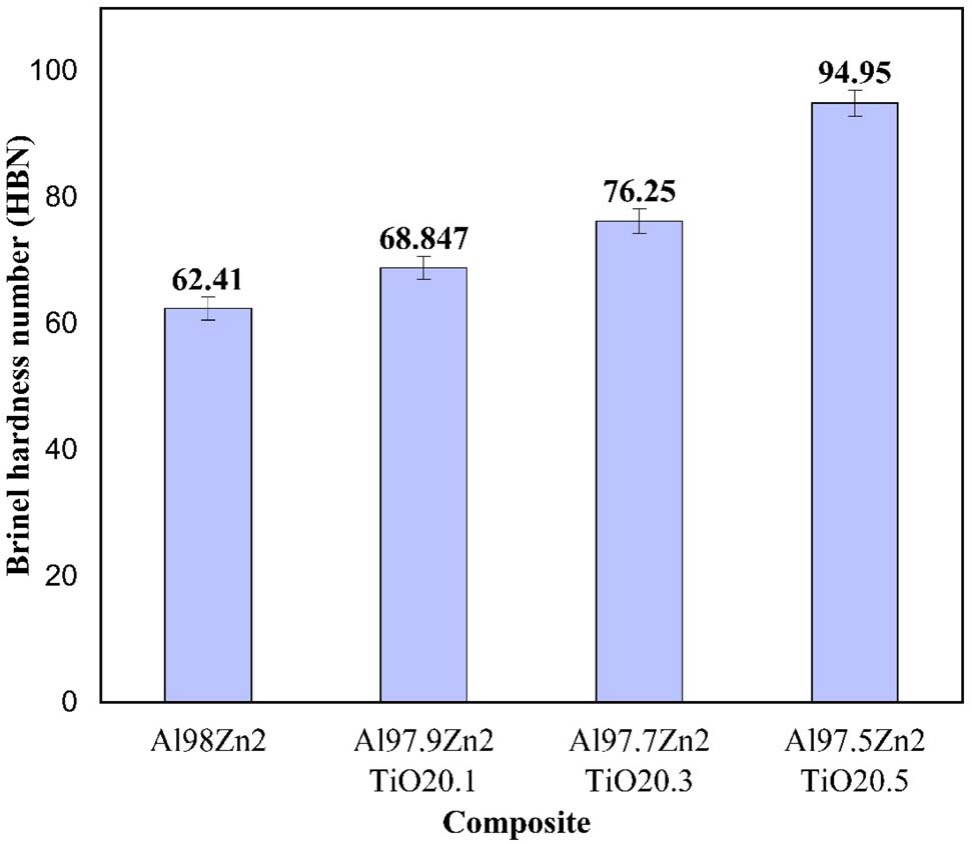
Hardness of different Al–Zn–TiO_2_ composites.

The Brinell hardness values for the Al-2 wt% Zn alloy and Al-2 wt% Zn-TiO_2_ composites are shown in Fig. 10. An increase in hardness with increasing TiO_2_ content indicates that the ceramic reinforcement effectively increases the matrix's resistance to indentation. Similar hardness improvements with TiO_2_-containing Al composites processed by stir casting have been reported in the literature.^84,85^ The unreinforced alloy (Al98Zn2) exhibits a hardness of 62.41 HBN. The addition of 0.1 wt% TiO_2_ increases hardness to 68.85 HBN (≈10.31% increase), while 0.3 wt% TiO_2_ yields 76.25 HBN (≈22.17% increase). The maximum hardness of 94.95 HBN is obtained at 0.5 wt% TiO_2_, corresponding to an overall improvement of approximately 52.14% relative to the base alloy. The relatively small standard deviation values confirm good measurement repeatability. The observed hardness enhancement is strongly supported by microstructural evidence. Optical micrographs (Fig. 2) show progressive grain refinement with increasing TiO_2_ content, leading to higher grain boundary density and Hall–Petch strengthening. SEM analysis (Fig. 4 and 5) further confirms the dispersion of TiO_2_/oxide-bearing particles within the α-Al matrix. These hard ceramic particles act as load-bearing constituents during indentation and impede dislocation motion through particle–dislocation interactions (Orowan strengthening). In addition, the reduction in void content with increasing TiO_2_ fraction (Table 1) indicates improved structural integrity, which enhances resistance to indentation. Overall, grain refinement, particle strengthening, and reduced porosity account for the significant increase in hardness, consistent with strengthening mechanisms reported for particulate-reinforced Al matrix composites.^86^

#### 3.2.4 Statistical analysis of mechanical properties

A one-way analysis of variance (ANOVA) was performed for each mechanical property—tensile modulus, tensile strength, impact strength, and hardness—to determine whether the mean values differ significantly among the four composite formulations. The composite type was treated as a categorical factor, and a significance level (*α*) of 0.05 was adopted. Table 3 summarizes the ANOVA results for each property, including the between-groups sum of squares (SS), degrees of freedom (df), mean square (MS), *F*-value, and *p*-value. The tensile modulus does not show a statistically significant difference among the composite samples. The calculated *F*-value for tensile modulus is 1.26 with a *p*-value of 0.321, which is greater than the significance threshold of 0.05. Therefore, the null hypothesis cannot be rejected for this property, indicating that the variation in tensile modulus among the composites is not statistically significant. In contrast, tensile strength demonstrates statistically significant differences among the samples, with an *F*-value of 78.86 and a *p*-value effectively equal to zero. Similarly, impact strength shows significant variation with an *F*-value of 127.14 and a *p*-value approaching zero. Hardness also exhibits a statistically significant difference among the composites, with a very high *F*-value of 312.43 and a *p*-value close to zero, indicating a highly significant variation among the samples. In these cases, the between-group variation is substantially larger than the within-group variation, leading to rejection of the null hypothesis of equal means. Overall, the one-way ANOVA analysis confirms that composite composition significantly affects hardness, impact strength, and tensile strength at the 95% confidence level, while its effect on tensile modulus is statistically insignificant.

**Table 3:** One-way ANOVA summary for the effect of composite formulation on mechanical properties

Property	SS (between)	df	MS (between)	*F*-value	*p*-value	Significance level (*α* = 0.05)
Tensile modulus (GPa)	5.449	3	1.816	1.26	0.321	Not significant
Tensile strength (MPa)	3160.3	3	1053.43	78.86	0	Significant
Impact strength (kJ m^−2^)	5783.3	3	1927.76	127.14	0	Significant
Hardness (BHN)	2972.12	3	990.705	312.43	0	Significant

## 4 Conclusion

This study shows that low TiO_2_ additions (0.1–0.5 wt%) influence the microstructure and mechanical behavior of stir-cast Al–Zn composites. Increasing TiO_2_ content promoted grain refinement, improved microstructural homogeneity, and reduced void content, while Zn remained predominantly in solid solution. At 0.5 wt% TiO_2_, the composite exhibited the highest hardness, impact strength, and tensile strength compared to the unreinforced alloy. Fractographic observations indicated a transition from predominantly ductile shear fracture in the matrix alloy to a mixed-mode fracture in the reinforced composite, consistent with the observed increase in strength and reduction in ductility. Overall, the results indicate that low TiO_2_ reinforcement can improve the strength-related properties of Al–Zn composites fabricated by stir casting under the present processing conditions.

## Author contributions

Md. Sabbir Hossain Shawon: writing – original draft, supervision, methodology, investigation, conceptualization. Aquib Rahman: writing – original draft, visualization, methodology, investigation. Chowdhury Ahmed Shahed: writing – review & editing, validation, supervision, methodology. Rezaul Karim Nayem: investigation, formal analysis. Rafia Islam: investigation, formal analysis. Asraf Ali Ratul: resources, data curation. Md Zillur Rahman: writing – original draft, validation, supervision, writing – review & editing.

## Conflicts of interest

The authors declared no potential conflicts of interest with respect to the research, authorship, and/or publication of this article.

## Data Availability

All data analyzed during this study are included in this article. No additional datasets were used.
